# Editorial: Advancements in research on plant-derived extracellular vesicles and nanoparticles- applications in biotechnology and one health

**DOI:** 10.3389/fbioe.2026.1900290

**Published:** 2026-06-18

**Authors:** Gabriella Pocsfalvi, Sandra Isabel Correia, Ani Barbulova

**Affiliations:** 1 Institute of Biosciences and BioResources, National Research Council (CNR), Napoli, Italy; 2 Centre for Functional Ecology, Department of Life Sciences, University of Coimbra, Coimbra, Portugal

**Keywords:** biotechnology, extracellular vesicle (EV), nanovescicles, one-health approach, plant

Plant extracellular vesicles (EVs) and plant-derived nanoparticles (PDNPs) have emerged as a rapidly evolving field bridging plant biology, nanotechnology, and translational science. This Research Topic was conceived to capture recent advances in this interdisciplinary area, highlighting both fundamental insights and emerging applications within biotechnology and the One Health framework.

Living plant cells actively secrete membrane-bound vesicles into the extracellular space, now recognized as plant EVs. These vesicles are formed through regulated intracellular trafficking pathways and carry molecular cargo, including RNAs, proteins, lipids, and metabolites. In contrast, PDNPs are typically isolated from disrupted plant tissues and represent heterogeneous populations of vesicular and non-vesicular nanoparticles originating from plant biomass. Although these entities are often grouped together, distinguishing between secretion-derived vesicles and disruption-derived nanoparticles is essential for improving reproducibility, functional interpretation, and standardization across studies.

The Research Topic of nomenclature remains central to the field. Current recommendations advocate the use of “extracellular vesicles” strictly for naturally secreted particles, while “plant-derived nanovesicles” or “nanoparticles” should describe materials obtained through mechanical disruption. This distinction is not merely semantic, but reflects fundamental differences in biogenesis, composition, and biological roles. Alongside nomenclature, methodological challenges persist. Isolation of plant EVs requires careful recovery from extracellular compartments such as apoplastic fluids or culture media, whereas PDNPs can be obtained at high yield from plant homogenates, albeit with increased heterogeneity.

Recent progress in purification strategies is beginning to address these limitations. Conventional approaches such as differential ultracentrifugation and density gradients are now complemented by more scalable technologies, including ultrafiltration, size-exclusion chromatography, and chromatographic platforms. These innovations are critical for improving particle purity and reproducibility, enabling more robust functional studies and facilitating translation toward industrial and biomedical applications.

Beyond methodological considerations, plant EVs play active roles in intercellular and cross-kingdom communication, particularly in plant defense mechanisms, where they deliver small RNAs and proteins to interacting organisms. In parallel, PDNPs have demonstrated significant functional relevance to human health, notably through the consumption of plant-derived foods in nutrition. Although PDNPs are not characterized by a defined biogenetic pathway, they act as naturally occurring nanocarriers enriched in bioactive lipids and metabolites. Their inherent stability, low immunogenicity, and scalability make them attractive candidates for applications ranging from drug delivery and immunomodulation to agriculture and nutrition.

The contributions collected in this Research Topic reflect these conceptual and technological advances and highlight the diversity of approaches currently shaping the field.

The mini-review by Alsaid et al. provides a comprehensive framework for understanding the fundamental distinctions between plant EVs and PDNPs, addressing their origins, molecular composition, and functional roles. The authors emphasize the importance of differentiating these systems, particularly in the context of cross-kingdom communication, where plant EVs are implicated in RNA-mediated interactions with pathogens, while PDNPs demonstrate strong translational potential in drug delivery, microbiome modulation, and immunotherapy. Importantly, this work also identifies critical challenges, including the lack of standardized biomarkers and the need for more consistent isolation methodologies.

Methodological innovation is further advanced by Zanotti et al., who present a scalable purification strategy that couples ultrafiltration with anion-exchange chromatography to isolate nanovesicles from *Brassica oleracea*. This approach enables the production of highly purified and homogeneous particle populations while preserving biological activity. Their multi-omics characterization reveals complex cargo composition, including proteins, lipids, and miRNAs, supporting a functional role in modulating inflammation and wound healing processes in human cells. This work represents a significant step toward industrial-scale production of plant-derived nanovesicles.

The study by Moubarak et al. explores the biological activity of PDNPs derived from *Ginkgo biloba* seeds, demonstrating their protective effects on endothelial cells under inflammatory conditions. These nanovesicles reduce pro-inflammatory cytokine expression and preserve endothelial integrity, highlighting their potential for vascular health applications. Notably, this study also exemplifies the value of seed-derived nanoparticles as an underexplored resource with distinct biochemical composition and functional properties.

Expanding the scope beyond higher plants, Szebeni et al. investigate extracellular vesicles derived from the cyanobacterium *Spirulina maxima*, illustrating the broader relevance of photosynthetic organisms as sources of bioactive vesicles. Their work demonstrates that these vesicles exhibit significant antifibrotic activity in both *in vitro* and *in vivo* settings, inhibiting TGF-β- and PDGF-driven profibrotic pathways and reducing peritoneal fibrosis in a mouse model. Mechanistically, the presence of conserved chaperone proteins such as HSP70 and PARK7 within the vesicle cargo suggests that evolutionarily conserved molecules may mediate therapeutic effects across biological systems. These findings highlight the potential of algae-derived vesicles—conceptually related to plant EVs—as scalable, biocompatible, and low-cost alternatives to mammalian extracellular vesicles.

Collectively, the articles in this Research Topic underscore the convergence of plant biology, nanotechnology, and biomedical research. They reveal both the mechanistic diversity and the translational potential of plant-derived nanovesicles while emphasizing the importance of methodological rigor and conceptual clarity.

These advances align closely with the One Health paradigm, which recognizes the interconnectedness of plant, animal, human, and environmental health. Plant EVs and related nanovesicles offer a unique platform for cross-kingdom communication, capable of modulating microbial communities, delivering bioactive compounds, and influencing host physiology. Their natural origin and sustainability further position them as environmentally friendly alternatives to synthetic nanomaterials, with potential applications across agriculture, food systems, and medicine.

Despite these promising developments, several challenges remain. Standardization of nomenclature and isolation protocols is urgently needed, alongside the identification of robust molecular markers for plant EVs. Recognizing these priorities, the recent establishment of the Plant EVs Task Force within the International Society for Extracellular Vesicles represents a timely and important step for the field. By bringing together researchers across disciplines, the Task Force aims primarily to promote experimental rigor and reproducibility, foster critical evaluation of methodologies, and support the exchange of best practices tailored to the unique characteristics of plant extracellular vesicles. Greater mechanistic understanding of vesicle biogenesis, cargo selection, and uptake pathways will also be essential to support reproducibility and translation. Addressing these challenges will be key to unlocking the full potential of plant-derived nanovesicles as reliable tools in biotechnology and health.

In conclusion, this Research Topic provides a comprehensive overview of current progress in the field while identifying key directions for future research. By integrating fundamental insights with emerging applications, it contributes to establishing a solid foundation for the development of plant EV and PDNP technologies within the broader context of One Health.

